# Distinct Glucocorticoid Receptor Actions in Bone Homeostasis and Bone Diseases

**DOI:** 10.3389/fendo.2021.815386

**Published:** 2022-01-10

**Authors:** Sooyeon Lee, Benjamin Thilo Krüger, Anita Ignatius, Jan Tuckermann

**Affiliations:** ^1^ Institute for Comparative Molecular Endocrinology, University of Ulm, Ulm, Germany; ^2^ Institute of Orthopedic Research and Biomechanics, Trauma Research Center Ulm, Ulm University Medical Center, Ulm, Germany

**Keywords:** glucocorticoid receptor, transgenic mice, osteoporosis, osteoblast, osteoclast

## Abstract

Glucocorticoids (GCs) are steroid hormones that respond to stress and the circadian rhythm. Pharmacological GCs are widely used to treat autoimmune and chronic inflammatory diseases despite their adverse effects on bone after long-term therapy. GCs regulate bone homeostasis in a cell-type specific manner, affecting osteoblasts, osteoclasts, and osteocytes. Endogenous physiological and exogenous/excessive GCs act *via* nuclear receptors, mainly *via* the GC receptor (GR). Endogenous GCs have anabolic effects on bone mass regulation, while excessive or exogenous GCs can cause detrimental effects on bone. GC-induced osteoporosis (GIO) is a common adverse effect after GC therapy, which increases the risk of fractures. Exogenous GC treatment impairs osteoblastogenesis, survival of the osteoblasts/osteocytes and prolongs the longevity of osteoclasts. Under normal physiological conditions, endogenous GCs are regulated by the circadian rhythm and circadian genes display oscillatory rhythmicity in bone cells. However, exogenous GCs treatment disturbs the circadian rhythm. Recent evidence suggests that the disturbed circadian rhythm by continuous exogenous GCs treatment can in itself hamper bone integrity. GC signaling is also important for fracture healing and rheumatoid arthritis, where crosstalk among several cell types including macrophages and stromal cells is indispensable. This review summarizes the complexity of GC actions *via* GR in bone cells at cellular and molecular levels, including the effect on circadian rhythmicity, and outlines new therapeutic possibilities for the treatment of their adverse effects.

## Introduction

Glucocorticoids (GCs) are steroid hormones that respond to stress and the circadian rhythm. Endogenous GCs are released by the adrenal glands upon activation of the hypothalamic-pituitary-adrenal (HPA) axis. Excessive or insufficient levels of endogenous GCs, Cushing's syndrome or Addison's disease, respectively, result in low bone mass and increased fracture risk (1–5). Due to their anti-inflammatory potential, exogenous GCs like dexamethasone, prednisolone, and many others are synthesized for pharmacological applications. Since the late 1940s they are widely used to treat autoimmune and chronic inflammatory diseases, recently they have been also utilized for Covid-19 treatment (6–8). However, long-term GC therapy can cause severe adverse effects in bone such as osteoporosis, and 30-50% of those patients experience fractures (9, 10).

Once GCs enter their target cell, they become activated by the 11β-hydroxysteroid dehydrogenase type 1 (11β-HSD1) or deactivated by 11β-HSD2 (11, 12). After that initial step, the activated GCs bind to the glucocorticoid receptor (GR), a member of the nuclear receptor superfamily. GR is ubiquitously expressed and acts as a monomer, homodimer or even a tetramer (7, 13). The ligand-bound GR translocates into the nucleus and induces transactivation or transrepression of target genes in several ways (7): 1) direct binding of GR homodimers or oligomers to DNA associated GC-response elements (GRE), 2) direct binding of GR monomers to GRE, 3) tethering as a GR monomer to other DNA-bound inflammatory transcription factors such as NF-κB, AP-1, IRF-3 or Stat3.

Despite this common mechanism of GCs *via* GR, endogenous and exogenous GCs act distinctively in bone and are dependent on pathophysiological environments. Thus, it is necessary to understand the role of GCs in bone cells and their mechanism of action in several bone diseases. This review summarizes the status of current studies on cellular and molecular, endogenous and exogenous GC actions *via* the GR in bone cells. Additionally, it describes the effect of circadian rhythmicity in GC actions, and outlines new therapeutic possibilities for the treatment of their adverse effects.

## Endogenous GC Action in Bone Homeostasis

Endogenous GCs directly regulate bone homeostasis *via* the GR in a cell-type specific manner.

Several animal models have proved that GC signaling in osteoblast-lineage cells is critical to maintain bone mass. The effect of inactivated GC signaling in mature osteoblasts and osteocytes was investigated by overexpression of 11β-HSD2, the responsible enzyme for GC inactivation. A 2.3 kb or 3.6 kb fragment of *Col1a1* promoter-driven overexpression of 11β-HSD2 (Col2.3-HSD2 or Col3.6-HSD2) reduced cortical and trabecular bone mass in mice, which suggests the importance of GC signaling in osteoblast-lineage cells to regulate bone mass (14–16). Interestingly, another mouse model blocking GC action in osteoblast-lineage cells by osteocalcin promoter-driven overexpression of 11β-HSD2 (OG2-11β-HSD2) did not show any alteration in the bone under normal physiological conditions (17). These discrepancies among different mouse models could be explained by determining the specific stages of osteoblast-lineage cells or investigating cell-type specific conditional knock-out mouse models. Notably, GR deficiency in mice using cre overexpression under the control of early committed osteoblast progenitor markers (Runx2 or Osx1) resulted in decreased bone mass (18, 19). Taken together, endogenous GC signaling in osteoblast-lineage cells is essential in bone mass regulation. However, osteocyte-specific endogenous GC action remains inexplicit.

Osteoclasts, another key cell type for bone mass regulation, are not affected by endogenous GC signaling. Osteoclastogenesis and bone formation were normal in mice with the GR deleted in osteoclast progenitor cells (GR^LysMCre^) (18). Osteoclast-specific overexpression of 11β-HSD2 using the tartrate-resistant acid phosphatase (TRAP) promoter (TRAP-HSD2) did not alter bone mass in mice (20). Collectively, endogenous GC signaling does not affect osteoclastogenesis under normal physiological conditions.

However, GCs have a profound effect on bone loss that is induced by a model of microgravity- the hindlimb unloading (HU), a model that was developed for simulating the environment of astronauts during space voyages. In this HU model, rodents showed an elevated endogenous corticosterone level (21), which led to a decreased bone mass due to decreased osteoblastogenesis, and increased apoptosis of osteoblasts and osteocytes (22). However, blocking of GC signaling in mature osteoblasts and osteocytes using Col2.3-HSD2 transgenic mice did not alter cortical bone mass in the HU model (22). Osteoclastogenesis and bone resorption were enhanced during HU due to enhanced receptor activator of nuclear factor-κB ligand (RANKL) production in osteocytes (22). This outlines the importance of endogenous GC signaling in mature osteoblasts and osteocytes, in response to mechanical loading.

## Excessive Exogenous GC Action in Bone and GC-Induced Osteoporosis

Long-term GC therapy is the most common cause of secondary osteoporosis, which leads to an increased risk of fractures (23, 24). In patients, exogenous GCs with doses higher than 2.5 mg for more than 3 months are shown to weaken bone quality (25). There is also clear evidence that exogenous GCs inhibit osteogenesis (6, 10). Bone marrow stromal cells (BMSCs) isolated from patients with corticosteroid-induced osteonecrosis showed impaired osteogenesis (26). Similarly, BMSCs isolated from a rat GIO model displayed decreased proliferation and osteogenic differentiation (27). Application of exogenous GCs *in vivo* suppressed proliferation and differentiation of osteoblasts and induced apoptosis of osteoblasts and osteocytes, resulting in a low bone mass (17, 18). This side effect could partially be rescued by leukemia inhibitory factor (LIF) treatment that activated Stat3, Mapk/Erk, and Akt signaling in GC-treated cells (28). Despite long-term exposure to high dose GCs, osteoblast lineage-specific GR deficient mice (GR^Runx2cre^) displayed normal bone formation and unaltered osteoblast and osteocyte numbers (18). This is corroborated by studies with GC inactivation in mature osteoblasts and osteocytes, using mice overexpressed 11β-HSD2 under the osteocalcin gene 2 (OG2) promoter (OG2-11β-HSD2). In these mice, GC-mediated increased apoptosis of osteoblasts and osteocytes is abrogated as well (17). These studies show that exogenous GC treatment leading to GC excess impairs osteoblastogenesis, the survival of osteoblasts, and osteocytes.

GCs affect the cross-talk among bone cells. Exposure to high doses of GCs results in an increased amount of RANKL secreted by osteoblasts and osteocytes. In turn, this increases the RANKL to osteoprotegerin (OPG) ratio and enhances bone resorption by osteoclasts (29–31).

Excessive GCs can also directly affect osteoclastogenesis (20, 32). During the initial phase of the therapy, GCs increase bone resorption by promoting osteoclast proliferation, osteoclast differentiation, and prolonging their life span (20, 33–35). However, the effect of long-term GC exposure on osteoclasts is still not entirely resolved. A few studies reported that long-term GC excess rather reduces osteoclast activity due to disrupted cytoskeleton of the osteoclasts (35, 36). However, several other studies addressed osteoclast apoptosis after long-term GC exposure (32, 34, 35, 37). Some studies showed that GCs reduce osteoclast apoptosis (34, 35), while others reported that GCs do not affect osteoclast apoptosis at all (32, 37). Collectively, pharmacological GCs affect osteoclastogenesis and bone resorption either directly, or *via* increased RANKL secretion from osteoblasts/osteocytes.

## GCs in Skeletal Stem Cells

Skeletal stem cells are essential for bone development, growth, and maintenance (38). During the last decade, skeletal stem cell markers have been identified in humans and rodents (38–41). To date, however, the role of GCs in these cells has not yet been extensively explored. Earlier, a study demonstrated that GR deletion in mesenchymal tissues using Dermo1-Cre induces postnatal lethality due to defects in the lung and intestines (42). GR silencing on human BMSCs showed an inhibited osteogenic differentiation *in vitro* (43). These studies imply that GC signaling *via* the GR plays a key role in mesenchymal stem cells (MSCs) differentiation towards osteoblasts.

It is also known that GIO is clinically described by decreased bone mass along with increased marrow adiposity (24), indicating that GR regulates the balance between osteoblastogenesis and adipogenesis of MSCs (44). High GC doses (1 µM Dexamethasone) increased adipogenesis of human BMSCs regulated by c-Jun signaling (43). Other studies suggested that GCs induce adipogenic regulators. Adipogenesis was promoted in cortisol (1 µM) treated mouse bone marrow-derived stromal cell line ST-2, by increasing expression of Peroxisome proliferator-activated receptor-gamma2 (PPAR-γ2) and CCAAT/enhancer-binding protein (C/EBP) transcription factors that are the adipocyte master regulator (45). Similarly, C/EBPalpha expression was increased in bone of dexamethasone-treated mice (50 mg/kg daily for 5 weeks) as well as in primary BMSCs isolated from those mice (46). Dexamethasone treatment in rat BMSCs also increased PPAR-γ expression in a dose-dependent manner, whereas a PPAR-γ knockdown promoted osteogenesis (47). This GC-induced PPAR-γ expression increases Secreted frizzled-related protein 5 (SFRP5) expression which inhibits the Wnt/β-catenin pathway and thus suppresses osteogenesis (47).

Taken together, endogenous GCs promote osteoblastogenesis of MSCs, whereas exogenous or excessive GCs regulate the balance between osteoblastogenesis and adipogenesis of MSCs. Further studies are necessary to investigate the role of GCs in the fate decision of skeletal stem cells *in vivo*.

## Circadian Rhythmicity and GCs

Endogenous GCs are released under the control of circadian rhythms, that are modulated by the central circadian clock in the suprachiasmatic nucleus (SCN) of the hypothalamus (48). The daily rhythmicity of plasma GC levels modulates physiological processes in many peripheral tissues including bone (48, 49).

Indeed, diurnal rhythm appears in some bone metabolic markers such as the bone resorption marker C-terminal cross-linked telopeptide of type I collagen (CTX), osteocyte function marker fibroblast growth factor 23 (FGF23), and turnover marker serum osteocalcin (50–53). Other bone markers such as sclerostin, procollagen type 1 N-terminal propeptide (P1NP), OPG, or soluble RANKL serum levels did not display rhythmicity (50, 52). However, the 24-hour serum profiles of men displayed that bone formation marker P1NP was significantly reduced after a long-term (3 weeks) disruption of the circadian rhythm despite no alteration of CTX level (54). In mice, disrupted circadian rhythm by weekly alternating light-dark cycles (10 or 15 weeks) led to a reduced level of both P1NP and CTX, implicating a decreased bone turnover due to disrupted circadian rhythm (55). This is likely due to the altered expression level of circadian locomotor output cycles kaput (*Clock*) genes that regulate the circadian rhythm in bone cells (55). Unexpectedly, unlike with the P1NP level, osteoblast surface increased in these mice (55). Together with decreased osteoclast surface, trabecular bone mass was increased in these mice despite altered *Clock* gene expression in the bone due to disrupted circadian rhythm (55). Nevertheless, this study indicated the importance of circadian rhythm in bone health. Investigations considering different ages and duration of circadian rhythm disruption would provide further insights into the effects of circadian rhythm in bone.

Furthermore, genetic deletion of *Clock* genes in mice leads to altered bone phenotypes (56–61). Under normal physiological conditions, brain and muscle aryl hydrocarbon receptor nuclear translocator-like protein 1 (*Bmal1*) and period 1 (*Per1*) genes are expressed with oscillatory rhythmicity in bone (58, 61). *Bmal1* knock-out mice and mice with *Bmal1* deletion in Osx+ osteoblast precursors and their progeny showed a decreased bone mass with increased bone resorption, suggesting that *Bmal1* regulates bone homeostasis by controlling osteoblast-mediated bone resorption (58). An osteoclast-specific *Bmal1* knock-out mouse showed an increased bone mass due to reduced osteoclast differentiation, indicating *Bmal1* also regulates osteoclast-mediated bone resorption (57). The *Clock* gene that forms heterodimers with *Bmal1* or *Bmal2* regulates bone formation *via* protein disulfide isomerase family A member 3 (*Pdia3*), shown by reduced bone formation and increased apoptosis of osteoblasts in *Clock* knock-out mice (56). On the other hand, physical stress-induced GC signaling induces only the *Per1* gene in mouse liver, heart, lung, and stomach by binding the GR to the GRE in the *Per1* promoter (62). However, it is not yet known if the GR directly binds to *Bmal1*, *Clock*, and *Per1* promoters to modulate their actions in bone cells.

Conversely, a single injection of synthetic corticosteroids can reset the circadian time in the periphery such as the liver, kidney, and heart by modulating circadian gene expression (63–65). Short-term dexamethasone treatment (2 hours) synchronizes circadian gene expression in osteoblast and osteoclasts *in vitro* (66, 67). Upon GC treatment, this circadian rhythm was also observed in cultured osteoblasts of Per1::luciferase transgenic mice (58). A single injection of dexamethasone could restore the circadian rhythm of osteoclast-related genes such as cathepsin K (*Ctsk*) in adrenalectomized mice (66). *Per2* knock-out mice could not restore the GC-induced bone loss despite a bisphosphonate (Zoledronic acid) treatment, although *Per2* knock-out osteoblasts showed an increased proliferation capacity (68).

However, constant GC exposure by inserting slow-release corticosterone pellets led to a shutdown of the endogenous HPA axis due to negative feedback, and thus to a flattening of GC-mediated circadian rhythm mediated gene expression (69). This resulted in bone loss not only by the excessive effects of GCs but also due to disrupted circadian gene expression, increased circulating bone resorption marker, and decreased bone formation (69).

Taken together, daily endogenous GC rhythm is important for bone homeostasis. A single treatment with exogenous GCs can regulate circadian gene expression, whereas disrupted circadian rhythm by continuous GC exposure contributes in addition to direct GC effects on osteoporosis.

## Influence of GCs on Bone Fracture Healing

It is well known that patients undergoing long-term GC medication are at a significantly increased risk for bone fractures (23, 70). Even though steroid use has not been found to be a major risk factor for non-union fracture healing in clinical studies (71), preclinical studies indicate that GCs also influence the complex fracture healing process (6, 72). This applies not only to GC therapy but also to endogenous GCs which control many physiological processes and, as stress hormones, are released upon a bone fracture. It can be anticipated that endogenous as well and exogenous or excessive GCs influence all stages of bone fracture healing, which necessitates a finely tuned interaction between multiple cell types, including immune, bone, and stromal cells which are all crucially regulated by GCs (6, 72). A fracture leads to the disruption of bone, blood vessels, soft tissues, and the release of danger-associated molecular patterns (DAMPS). These quickly trigger an innate immune response to contain the damage, and clear the wound site from tissue debris and pathogens (73–75). The initial response involves the activation of the complement system, the release of inflammatory chemokines and cytokines from local immune, endothelial and mesenchymal cells, as well as the recruitment and activation of further immune cells, mainly neutrophils, monocytes, and macrophages. Later, lymphocytes are also recruited to the fracture site and initiate an adaptive immune response. The inflammatory phase is regarded to promote the recruitment, proliferation, and differentiation of mesenchymal and endothelial precursor cells, which are essential for subsequent healing processes. This process comprises of the formation of a soft callus with fibrous and cartilaginous tissue, which is then continuously transformed into the bone by endochondral ossification. Finally, the hard callus is remodeled until the original bone structure is restored (73–75).

So far, only a few studies have addressed the role of endogenous GCs during fracture healing by using mouse models with impaired GC signaling (76–79). Fracture healing was significantly impaired when the endogenous GC action was globally eliminated by using mice with an inducible GR knock-out (GR^gtROSACreERT2^) (78). In these mice, the early systemic and local immune responses upon fracture were significantly increased. During callus formation, cartilage-to-bone transformation was disturbed, confirmed by persisting cartilage and reduced bony bridging of the fragments in GR^gtROSACreERT2^ mice. This study suggests a crucial role of endogenous GCs in all stages of fracture healing. Several studies showed the role of GC signaling in distinct cell types during bone regeneration. When GC signaling was disrupted in osteoblasts using Col2.3-11ß-HSD2 mice (76), intramembranous bone formation was not affected, whereas GR deletion in chondroblasts using GR^Col2CreERT2^ mice resulted in impaired endochondral bone healing, by increasing the cartilaginous fraction of the fracture callus (77). To investigate whether GR dimerization (which is regarded to be essential for the anti-inflammatory effects of GCs) is important for fracture healing, Hachemi et al used mice with a defective GR dimerization ability (GR^dim^) (79). Impaired GR dimerization had no significant effect on the healing process in a model of isolated femur fracture (79). However, in a model of compromised fracture healing, induced by hyperinflammation in a combined model of fracture and thoracic trauma, impaired GR dimerization in GR^dim^ mice reduced inflammation and abolished the deleterious effects of posttraumatic hyperinflammation on fracture healing (79). In summary, these studies demonstrate that endogenous GCs promote fracture healing by controlling the immune response and by stimulating cartilage-to-bone transition.

In contrast to endogenous GCs, exogenously applied GCs can provoke negative effects on the fracture healing process as demonstrated in pre-clinical investigations in different species, including rabbits (80, 81), rats (82), and mice (83, 84). Consistently, these studies report impaired cartilage-to-bone transformation, reduced quality and structure of the newly formed bone, and poor biomechanical properties of the fracture callus. However, these studies are mostly descriptive and the molecular and cellular reasons for the delayed bone healing under long-term GC therapy are still not fully understood.

## GCs action in Rheumatoid Arthritis

Although GCs are used to ameliorate the symptoms of rheumatoid arthritis (RA) since the 1950s, there are still surprises concerning the mode of action of GCs, their activating enzymes 11β-HSD1 and the GR requirement in distinct cell types. In RA and osteoarthritis, GCs are still in frequent use, in combination with other treatment regimens (85). Preclinical animal models for the GC modulating enzyme 11β-HSD1/2 and the GR in distinct cell types revealed distinct requirements of GC function in different cells depending on the model. First of all, the attenuation of complete GR dimerization by a knock-in of a point mutation into the second zinc finger demonstrates that an intact function of the GR allows gene regulation beyond the suppression of cytokines in different RA models (86, 87). Accordingly, global inhibition of the GC activating 11β-HSD1 abrogated the therapeutic response towards corticosterone by reduction of inflammatory symptoms in mice (88).

However, the definition of critical cell types for mediating GC action present in RA varied in distinct animal models. In antigen-induced arthritis, GR in T cells (presumably in Th17 cells) was critical to confer anti-inflammatory effects, since mice lacking the GR in T cells were completely resistant to the dexamethasone-mediated reduction of joint swelling (86). In serum transfer-induced arthritis, however, there was the surprising discovery that global GR deletion in hematopoietic cells by hematopoietic stem cell transfer into irradiated wild-type mice did not abrogate the therapeutic effects of dexamethasone (87). Vice versa GR global knock-out mice and mice with attenuated GR dimerization failed to respond to dexamethasone, even when their hematopoietic system was reconstituted by GR wild-type cells (87). These mice could not induce anti-inflammatory macrophages in the joint which are critical to resolve inflammation in RA (87). Elimination of the GR in fibroblasts (Col1a2CreERT2) attenuated the therapeutic response, strongly suggesting that GCs affect the fibroblast like-synovial cell (FLS) – macrophage crosstalk *via* the GR (87). Intriguingly, Hardy and colleagues showed that GC production in myeloid cells might be necessary for re-activating GC function (88). Thus, cellular cross-talk targeted by systemic and locally produced GCs seems to underly the therapeutic actions of GCs, which need to be further elucidated. Given that FLS exists in pro-inflammatory and anti-inflammatory subsets (89, 90) and interstitial/lining macrophages are existing with different fates in arthritis (91), this raises the complexity and fine-tuning of GR cross-talk.

## Conclusions and Perspectives

**Figure 1:**
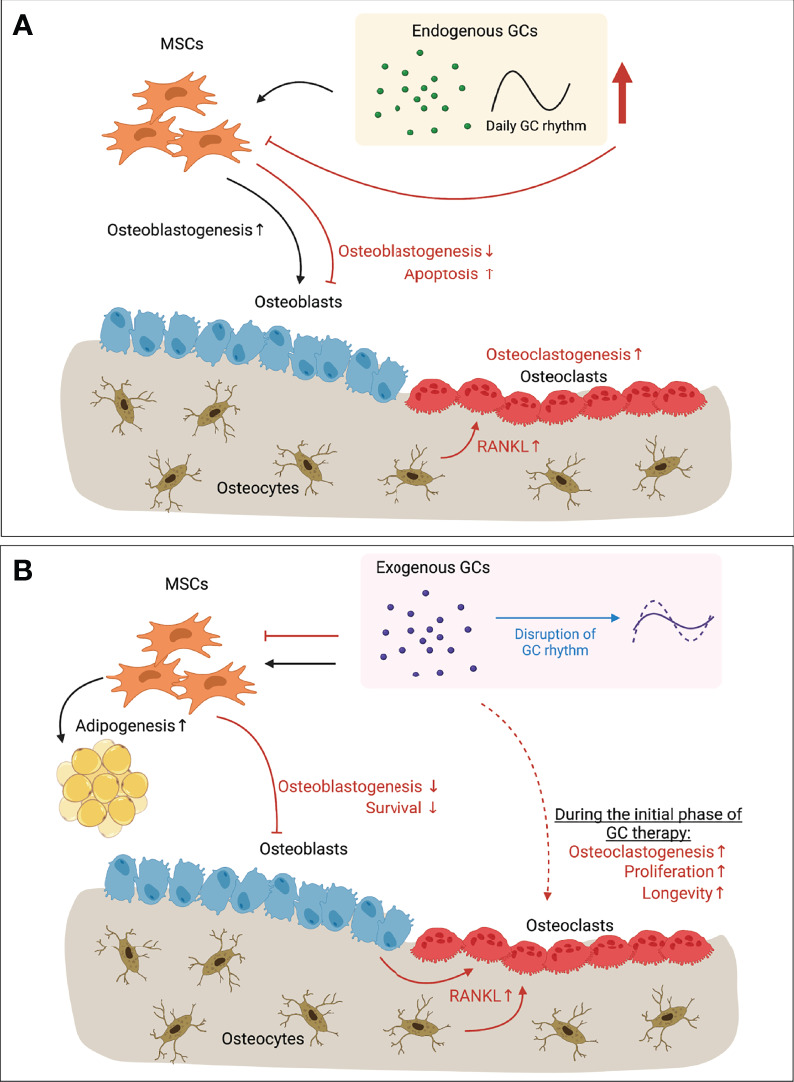
Paradoxical effects of GCs in bone. **(A)** Endogenous GCs regulated by circadian rhythm (and expressing daily GC rhythm accordingly) have anabolic effects on osteoblastogenesis (black arrows). When endogenous GC level is increased upon stress (e.g. mechanical unloading), however, bone mass is decreased due to inhibited osteoblastogenesis, increased apoptosis of osteoblasts and osteocytes, and enhanced osteoclastogenesis due to the increased RANKL secreted by apoptotic osteocytes (red arrows). **(B)** Long-term exogenous GC therapy inhibits osteoblastogenesis and survival of osteoblasts (red arrows). Increased RANKL secretion by osteoblasts and osteocytes let enhance bone resorption by osteoclasts (red arrows). Direct action of exogenous GCs on osteoclasts has showed with increased osteoclastogenesis, increased proliferation and longevity of osteoclasts during the initial phase of GC therapy (dotted red arrow). However, direct effects of long-term GC therapy on osteoclasts still remain elusive. Exogenous GCs also regulate the balance between osteoblastogenesis and adipogenesis of MSCs that is one of feature of GIO (black arrows). On the other hand, continuous exogenous GC therapy can flatten the endogenous GCs rhythm (blue arrow), resulting in disrupted circadian gene expression and levels of circulating bone turnover markers. Together, Long-term GC therapy leads to bone loss by its direct action on bone cells, and/or via disrupting GC rhythm. GC, Glucocorticoid; RANKL, Receptor activator of nuclear factor-κB ligand; MSC, Mesenchymal stem cell; GIO, GC-induced osteoporosis. This illustration was created with BioRender.com.

GCs are frequently used drugs in clinics despite their detrimental effects on bone after long-term use. They act in cell-type specific manner, and *via* cellular crosstalk mechanisms, which are still partially unknown. Currently, some drugs are applied to treat GIO by either inhibiting osteoclast activity (Bisphosphonates and Denosumab) or stimulating osteoblast activity (Teriparatide) (92). However, the utilization of drugs to treat unwanted effects caused by other drugs is not ideal for patients. Thus, it is of utmost importance to develop new therapies with a cell-type specific delivery of GCs, and/or targeting downstream molecules to avoid or minimize the detrimental effects. Further understanding of the controversial effects of endogenous and exogenous/excessive GCs on the bone that are both anabolic and catabolic will help to develop therapeutic concepts (**Figures 1A, B**). Daily GC rhythm should be considered during GC therapy. Chronotherapy when administering GCs could help to increase therapeutic efficacy, and reduce detrimental effects, although further investigations are required considering that the drug half-life and bioavailability can be inflexible (93). In addition, preclinical models considering factors such as physical stress, aging, and diseases can be introduced to investigate diverse clinical settings. Advanced technologies such as single-cell RNA sequencing and lineage-tracing animal models will allow us to map the alteration of specific cell types present in bone in response to GCs. It will be also helpful to determine dynamic spatial profile and crosstalk among bone cells in clinically relevant models such as fracture healing and RA. Further studies are needed to understand how GC rhythm affects such disease models. These actions in combination will ultimately broaden our scope to approach innovative therapies.

## Author Contributions

SL, JT, BK, and AI wrote chapters of the article. All authors contributed to the article and approved the submitted version.

## Funding

This work is supported by the following funding resources: German Research Foundation (DFG) Tu220/14-1, DFG (Ci 216/2-1), and DFG in the framework of Collaborative Research Center CRC1149 "Danger Response, Disturbance Factors and Regenerative Potential after Trauma" (251293561-CRC 1149, INST 40/492-1, and INST 40/492-2).

## Conflict of Interest

The authors declare that the research was conducted in the absence of any commercial or financial relationships that could be construed as a potential conflict of interest.

## Publisher’s Note

All claims expressed in this article are solely those of the authors and do not necessarily represent those of their affiliated organizations, or those of the publisher, the editors and the reviewers. Any product that may be evaluated in this article, or claim that may be made by its manufacturer, is not guaranteed or endorsed by the publisher.
